# Enhanced Electrochemical Performance of Metallic CoS-Based Supercapacitor by Cathodic Exfoliation

**DOI:** 10.3390/nano13081411

**Published:** 2023-04-19

**Authors:** Ye Tian, Yuxin Ma, Ruijin Sun, Weichao Zhang, Haikun Liu, Hao Liu, Libing Liao

**Affiliations:** 1 School of Science, China University of Geosciences, Beijing 100083, Chinasunruijin@cugb.edu.cn (R.S.); 2 Beijing Key Laboratory of Materials Utilization of Nonmetallic Minerals and Solid Wastes, National Laboratory of Mineral Materials, School of Materials Science and Technology, China University of Geosciences, Beijing 100083, China; 3 National Center of Technology Innovation for Display, Guangdong Juhua Research Institute of Advanced Display, Guangzhou 510525, China

**Keywords:** two-dimensional nanomaterials, metallic cobalt sulfide, cathodic exfoliation, hybrid supercapacitor

## Abstract

Two-dimensional nanomaterials hold great promise as electrode materials for the construction of excellent electrochemical energy storage and transformation apparatuses. In the study, metallic layered cobalt sulfide was, firstly, applied to the area of energy storage as a supercapacitor electrode. By a facile and scalable method for cathodic electrochemical exfoliation, metallic layered cobalt sulfide bulk can be exfoliated into high-quality and few-layered nanosheets with size distributions in the micrometer scale range and thickness in the order of several nanometers. With a two-dimensional thin sheet structure of metallic cobalt sulfide nanosheets, not only was a larger active surface area created, but also, the insertion/extraction of ions in the procedure of charge and discharge were enhanced. The exfoliated cobalt sulfide was applied as a supercapacitor electrode with obvious improvement compared with the original sample, and the specific capacitance increased from 307 F∙g^−1^ to 450 F∙g^−1^ at the current density of 1 A∙g^−1^. The capacitance retention rate of exfoliated cobalt sulfide enlarged to 84.7% from the original 81.9% of unexfoliated samples while the current density multiplied by 5 times. Moreover, a button-type asymmetric supercapacitor assembled using exfoliated cobalt sulfide as the positive electrode exhibits a maximum specific energy of 9.4 Wh∙kg^−1^ at the specific power of 1520 W∙kg^−1^.

## 1. Introduction

Two-dimensional (2D) materials including graphene, graphene oxide (GO), layered transition metal dichalcogenides (LTMD), black phosphorus, hexagonal boron nitride and MXene have recently drawn tremendous attention since their discovery for their unique properties, versatile functionalities and potential applications [1,2,3]. Among them, the LTMD family of materials with distinctive physical and chemical properties has become a research hotspot for diverse applications, including energy storage and conversion and so on [4]. Most 2D layered materials exist in nature or can be readily synthesized, and when the thickness of the bulk multilayer solids is reduced to single or few layers thickness, they will possess extraordinary and useful properties that can be exploited in optical, catalytic or electronic applications [5]. Specifically, the outstanding features of 2D LTMD include highly exposed active edge positions, large specific surface area and easy insertion of other guest materials. In addition, the ultra-thin thickness results in LTMD possessing the advantage of higher specific surface area than their bulk counterparts, which makes them promising for applications in energy storage and conversion, for example, solar cells, electrocatalysis, rechargeable batteries and supercapacitors [6]. As an important member of LTMD, various stoichiometric cobalt sulfides such as CoS_2_, Co_8_S_9_, Co_3_S_4_, CoS are extensively found, which have been targeted because of their low cost and long life. It is a versatile material with potential applications in electrocatalysts, lithium-ion batteries, supercapacitors, alkaline rechargeable batteries, magnetic materials and solar cells [7,8]. To be specific, cobalt sulfide is considered a potential supercapacitor electrode candidate because of its intrinsically multiple valence states of Co atoms, diverse morphologies and high reversible redox activity. The biggest drawbacks restricting the application of its electrochemical performance, though, are its low electrical conductivity and slow ion transport kinetics compared to carbon materials [9,10].

Up to now, cobalt sulfide with various microstructures has been investigated as a new type of energy material exhibiting good electrochemical properties. For instance, Co_9_S_8_ nanoparticles were synthesized by the solvothermal techniques in a mixed solvent of cobalt diethyldithiocarbamate as single-source precursor and hexadecylamine as a shape-directing agent. Co_9_S_8_ nanoparticles modified electrode exhibits the specific capacitance of 502 F∙g^−1^ at the current density of 1 A∙g^−1^ in a 1 M KOH electrolyte [8]. Flower-like CoS was obtained from the purified leaching agent of LiCoO_2_ and the material can exhibit supercapacitor behavior with the maximum specific capacity for 409.3 mAh∙g^−1^ measured by 1 A∙g^−1^ [11]. Although CoS materials have been reported by various synthetic methods including microwave irradiation, chemical deposition and sol-gel method, it is difficult to synthesize high-purity cobalt sulfide with well-controlled morphology in the absence of templates and surfactants, and other complex phases such as cobalt oxide or cobalt hydroxide tend to form and are hard to remove due to the strong affinity of cobalt ions for oxygen. Consequently, finding an efficient and environmentally friendly method of preparing high-performance CoS-based supercapacitors remains a challenge. Additionally, most CoS presented a hexagonal densely packed wurtzite crystal structure that is not conducive to charge transfer. It has been reported that the novel metallic layered CoS crystal possesses high electrical conductivity and potential in the area of energy storage, but its capacitive performance has not been investigated [12,13,14].

As a typical 2D LTMD material, the structure of metallic layered CoS is analogous to graphite in that the atoms within a plane are bonded by strong bonds, while the adjacent layers are in contact through much weaker van der Waals force. In this case, the layers can slide in the in-plane direction under shear or expand in the out-plane direction due to the intercalator-assisted peeling process to produce less-layered structure. Currently, a variety of research interests have been focused on the methods for the exploration of exfoliating LTMD to single- or few-layer thin sheets by external stimulation, including mechanical shearing, ultrasonication and dispersion in liquid medium and electrochemistry. It is worth mentioning that the imposed driving force of the top-down approach determines the purity and quantity of the exfoliating products and the speed of the exfoliation process, including one- or multi-step exfoliating methods [15,16], in which mechanical shearing is one of the most common methods with high-quality products, but its practical application is hampered by the disadvantages of low efficiency that cannot be mass produced and hard-to-repeat experimental results. A relatively simple ultrasound-assisted exfoliation method, which is to directly treat bulk crystals with ultrasound in solvents or polymer/surfactant solution, has a weakness in producing LTMD nanosheets with small transverse sizes and relatively low yields [6]. All these deficiencies result in inefficiencies and high costs of the exfoliation production that prevent the practical production and application of 2D materials beyond the laboratory level. Alternatively, electrochemical intercalation or exfoliation has become an efficient and moderate method for the mass preparation of 2D nanosheets with the characteristic of efficient, relatively high product purity, and it operates at ambient conditions. This method typically inserts foreign molecules or charged ion species (i.e., anions or cations) between layers of bulk materials motivated by a stationary potential or electrical current to expand the interlayer spacing and weaken the interlayer forces, facilitating the detachment and collection of 2D nanosheets from solution [17]. The working electrode for electrochemical intercalation serves as either an anode or cathode corresponding to the oxidation intercalation anions and reduction intercalation cations. Typical cations available include alkali metal ions (Li^+^, Na^+^, K^+^), quaternary amines (TBA^+^) and organic ions (BMP^+^), while anions include sulfates (SO_4_^2−^), hydroxides (OH^−^) and halides (Cl^−^, Br^−^, I^−^), among others. The anodic exfoliation method is more commonly used on account of higher intercalation and expansion efficiency, while the cathodic exfoliation method is preferred because the absence of strong oxidation conditions and avoids unnecessary chemical phase transition that may occur during the anodic exfoliation process [18]. Although the intercalation efficiency of cations is relatively low, the reduction reaction occurring at the cathode helps enlarge the layer spacing, thus obtaining a large lateral size and high purity 2D materials. When a suitable voltage is applied over the working electrode and the counter electrode, the ionic species will be inserted into the interlayer causing dramatic structural expansion of the host electrode to achieve the effect of exfoliating bulk materials.

In this work, we demonstrated that 2D exfoliated CoS nanosheets (EF-CoS) can be prepared by a straightforward and low-cost electrochemical cathodic exfoliation of the metallic layered CoS bulk crystal (bulk-CoS). In the present work, metallic layered cobalt sulfide is employed for the first time in supercapacitor electrode material. Due to its outstanding electrical conductivity, the CoS powder sample, placed into a conductive carbon cloth without any conductive or binder additives as the working electrode, can be exfoliated to few-layer thickness nanosheets in an inexpensive 0.5 M Na_2_SO_4_ electrolyte solution under ambient conditions avoiding lengthy operations with dangerous organic solvents. As a supercapacitor electrode material, EF-CoS had a 1.47-fold increase in specific capacity up to 450 F∙g^−1^ over bulk-CoS (307 F∙g^−1^) at the current density of 1 A∙g^−1^ under identical conditions, as well as excellent capacity retention rate and good cycling performance. It has been investigated that the increased specific surface area, more abundant active sites, enhanced charge transfer characteristics and more contribution of diffusion processes were supposed to be the primary factors to improve the electrochemical properties of exfoliated CoS flakes. Therefore, the present work successfully fulfills the need for the economical, environmentally friendly and large-scale preparation of metallic 2D CoS with good electrochemical performance as supercapacitor electrode material.

## 2. Experimental

### 2.1. Preparation

**Figure 1:**
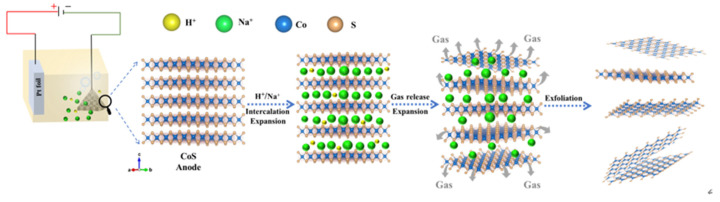
The schematic diagram of the electrochemical cathode exfoliation of layered CoS bulk crystal.

Bulk CoS was synthesized according to the reported method [14]. Briefly, the self-flux technique is initially used to develop numerous KCo_2_S_2_ single crystals in a typical run. After that, in a stainless steel autoclave with a Teflon liner of 25 mL volume, a suitable quantity of selenourea, sulfurea, iron powder and fragments of KCo_2_S_2_ single crystals were combined with 5 mL of deionized water. After the autoclave was heated at 100–120 °C for 96 h, single crystals with silvery metallic luster were collected. The experimental setup for electrochemical exfoliation is illustrated in Figure 1. With 100 mg CoS powder (average particle diameter of 0.2 mm) wrapped in carbon cloth as the cathode electrode and 1 × 1 cm platinum sheet as the anode electrode, the two electrodes immersed in 0.5 M Na_2_SO_4_ solution were placed at the distance of 3 cm in parallel. The bulk CoS powders were electrochemically exfoliated at the cathode electrode at the bias voltage of −2 V for 10 min followed by −15 V sustained for 1 h. Finally, the obtained electrolyte was further sonicated in a water bath for 3 h, centrifuged at 1500 rpm and collected by washing with ethanol and deionized water, then freeze-dried for 24 h to obtain EF-CoS.

### 2.2. Sample Characterization

The morphology of the prepared samples was investigated by scanning electron microscope (SEM, Gemini SEM 500) and transmission electronic microscope (TEM, FEI Tecnai Spirit) in which the elemental composition was detected using the EDAX octane Prime Energy Dispersion X-ray Spectrometer (EDX, Oxford X-MAX65T). X-ray diffractions (XRD, Cu Kα radiation, λ = 1.5406 Å, Bruker AXS D8 Advance) were utilized to examine the phase composition and crystal structure. The size and thickness of exfoliated CoS were determined by atomic force microscopy (AFM, Dimension Edge) of bright-field mode. The Brunauer–Emmett–Teller (BET) surface area of the samples was determined by ASAP2020 at ~77 K and calculated at isothermal points of relative pressure of P/P_0_ = 0.05–0.2.

### 2.3. Electrochemical Evaluation

For the supercapacitor measurements, the active slurry was obtained via admixing EF-CoS, binder poly(vinyl difluoride) (PVDF) and acetylene black in 1 mL of N-Methylpyrrolidone (NMP) solvent at a wt% of 8:1:1.The prepared slurry was well mixed and applied evenly on a carbon cloth size (1 × 2 cm^2^) and dried. First, the performance of the samples was investigated within 6 M KOH aqueous electrolyte solution by a conventional three-electrode system, where the working electrode, counter electrode and reference electrodes were the prepared electrodes, platinum sheets (1 × 1 cm^2^) and Hg/HgO electrode, respectively. Regarding the two-electrode system, a button-type asymmetric supercapacitor (ASC) was assigned in 1 M KOH aqueous electrolyte solution with the EF-CoS as the positive electrode and activated carbon (AC) as the negative electrode. Optimal performance was obtained by controlling the charge balance q_+_ = q_−_ of the two electrodes. The charge stored on each electrode can be calculated by Equation (1) below, and the optimal mass ratio of positive and negative loaded active substances was determined according to Equation (2):

(1) q=CmΔV

(2) $$\frac{m^{+}}{m^{-}}=\frac{C_{-}\Delta V^{-}}{C_{+}\Delta V^{+}}$$

where *m*, *C*, *ΔV* represent the mass, specific capacitance and potential window of the active material, respectively.

Additionally, the specific capacitances (*C_s_*), energy density (*E*, Wh∙kg^−1^) and power density (*P*, W∙kg^−1^) can be calculated using the following equation from the collected GCD curve [19]:

(3) $$C_{s}=\frac{I\times \Delta t}{3.6\times m}$$

(4) $$E=\frac{C_{s}\times \Delta V^{2}}{2}$$

(5) $$P=\frac{3600E}{\Delta t}$$

in which *C_s_* (mAh·g^−1^) is the specific capacitance, *I* (A) is the discharging current, *Δt* (s) represents the time for full discharging and *m* (g) expresses the total mass of the active materials on the working electrodes. The electrochemical measurements consisting of cyclic voltammetry (CV), galvanostatic charge–discharge (GCD) and electrochemical impedance spectrometry (EIS) were conducted using a work station (CHI760E), and cycling stability was performed using a Land CT3001A battery test kit. All electrochemical measurements were done at ambient temperature.

## 3. Results and Discussion

### 3.1. Characterization

The experimental setup and mechanism diagram of the CoS exfoliation process in sodium sulfate solution is shown in Figure 1. The bulk-CoS crystal in carbon cloth was used as the working electrode and the Pt sheet served as the counter electrode. The CoS sample used for exfoliation is a novel tetragonal phase shown in Figure 1, where each Co atom stands in the central position of the tetragonal coordination and is bound to four sulfur atomic ligands. Each sulfur center is linked to three cobalt centers through a pyramidal connection. The cobalt atoms are sandwiched between layers of sulfur atoms in the layered structure made of linked tetrahedra [20]. This layered CoS showed a high electronic conductivity of 5000 Ω^−1^∙cm^−1^ at room temperature. This peculiar large-scale interlayer structure is a favorable structure for ion embedding. Therefore, CoS nanosheets could be anodized from the bulk powder samples in aqueous electrolyte by introducing a package made of carbon cloth that is both electrically conductive and sufficiently in contact with the material. The overall electrifying procedure was applied to the working electrode with an initial low negative bias of 2 V to wet the bulk CoS crystals, then the bias voltage was increased to −15 V for 1 h to exfoliate the crystals. As a consequence, many CoS flakes were separated from the bulk crystals and diffused out of the carbon cloth to be suspended in the solution, and the electrolyte gradually deepened to constant with increasing time. The process mainly includes the flowing steps as shown in the right of Figure 1. First, the initial low pressure may lead to the reduction of water to generate hydrogen radicals assembled around the bulk CoS crystal. Then, bulk CoS as a negative electrode attracts cations including Na^+^ and H^+^ in the electrolyte intercalation, forming intercalated compounds Na_X_CoS to expand the spacing between crystal layers, which weakens the interlayer van der Waals force effectively. Next, the reduction of cations between the layers leads to the release of H_2_ at a sufficiently high voltage and the expansion of the CoS interlayer, resulting in significantly weakened interlayer attraction, triggering the intercalation of more cations to separate the flakes from the bulk crystal. Subsequent ultrasonic steps help further peel the bulk-CoS into thinner sheets and this process is performed under ambient circumstances and can also be expanded to produce large numbers of nanosheets [21,22].

**Figure 2:**
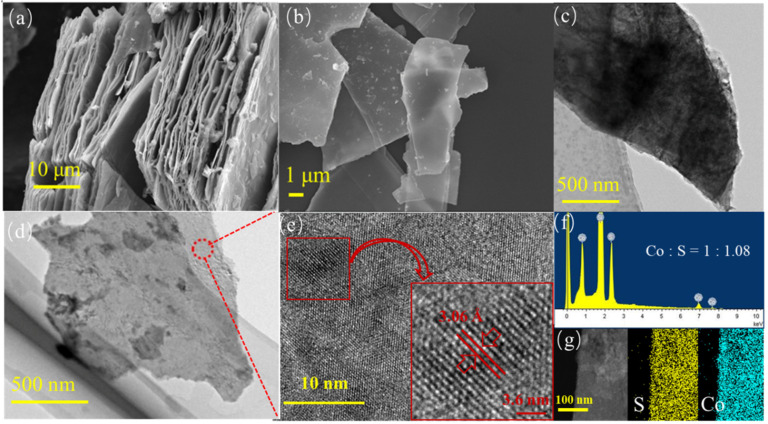
SEM images of bulk-CoS (**a**) and EF-CoS (**b**); TEM images of bulk-CoS (**c**) and EF-CoS (**d**); HRTEM micrographs (**e**), EDS results (**f**) and corresponding elemental mapping (**g**) of EF-CoS.

The morphology of bulk-CoS and EF-CoS was observed by typical SEM images in Figure 2a,b. In comparison with pristine bulk CoS in Figure 2a exhibiting the large lateral size and thicker structure of 50 μm, the CoS after 30 min of electrochemical exfoliation in Figure 2b has a smaller lateral size of 5–20 μm and a significantly thinner sheet, indicating that the bulk CoS particles are essentially fragmented and efficiently exfoliated. The relatively thin and thicker flakes of the samples can be easily distinguished by their different contrast using TEM, as shown in the low magnification TEM images of EF-CoS in Figure 2d. The contrast of EF-CoS with a transverse dimension of about 1 μm is significantly weaker than bulk-CoS of Figure 2c corroborating the lesser number of layers in the exfoliated sample. The high resolution TEM (HRTEM) image of Figure 2e exhibits clear lattice fringes with a crystal spacing of 3.06 Å. The EDS elemental scanning of Figure 2f taken on the nanosheets evidences the molar ratio of Co to S is close to 1:1, and the EDS elemental mapping image shows uniform distribution of Co and S elements in EF-CoS. In brief, the above results indicate that the obtained EF-CoS nanosheets have few defects and high crystallinity with intrinsic structure [23].

**Figure 3:**
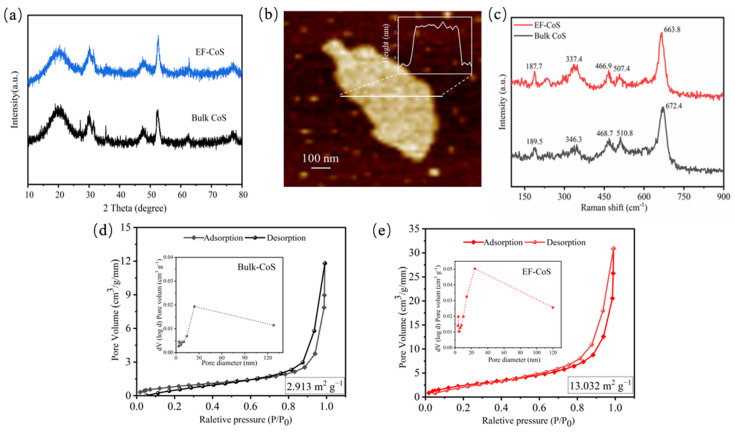
(**a**) XRD patterns comparison of bulk-CoS and EF-CoS; (**b**) AFM image of the topography of EF-CoS and corresponding height profile; (**c**) Raman spectra of AFM image of bulk-CoS and EF-CoS; (**d**,**e**) Nitrogen adsorption–desorption isotherms and cumulative pore volume of bulk-CoS and EF-CoS.

Figure 3a compares the XRD pattern of bulk-CoS and EF-CoS. The diffraction peaks of EF-CoS are almost retained and displayed lower reflection degree and broader diffraction peaks, suggesting that the crystallinity was still retained and the phase did not change during exfoliation. The distance between layer and layer (d-spacing of (002)) of EF-CoS is 0.44 nm according to the calculation for the corresponding peak reflection. As depicted in the Figure 3b, the AFM image of EF-CoS and corresponding scanning thickness profiles (inset in Figure 3b) demonstrate a typical flakes-like morphology whose height can be determined to be 3.0 nm, indicating the CoS nanosheet is composed of 8 layers given that the thickness of single-layered CoS is about 0.44 nm [24]. The Raman spectra of the samples before and after exfoliation were studied, as shown in Figure 3c. The Raman spectral pattern is consistent with the previously reported results of cobalt sulfide [25]. Compared with Bulk CoS, the Raman spectra of EF-CoS shifted to lower wavenumbers due to the increase in long-range Coulomb interactions between Co atoms with increasing interlayer spacing. Additionally, the increase in Raman spectral intensity is considered to enhance the conductivity of material, which is beneficial for its electrochemical energy storage performance. Additionally, the specific surface area was further performed by a nitrogen adsorption–desorption isotherm curve and estimated using the BET method to compare the changes before and after exfoliation. The results demonstrate the specific surface area of bulk CoS (2.913 m^2^∙g^−1^) is much smaller than that of CoS after exfoliation (13.032 m^2^∙g^−1^), demonstrating that EF-CoS has larger specific surface area to provide a larger active area for the interfacial reactions. Two effects of electrochemical exfoliation can be observed from the results of morphology: (1) large pieces of massive cobalt sulfide are broken into smaller sized flakes; (2) thick multilayer structures are separated into thinner few-layered flakes. The higher surface area provides more electrochemical active sites, which facilitates mass transport in the electrochemical energy storage process.

### 3.2. Electrochemical Performance

**Figure 4:**
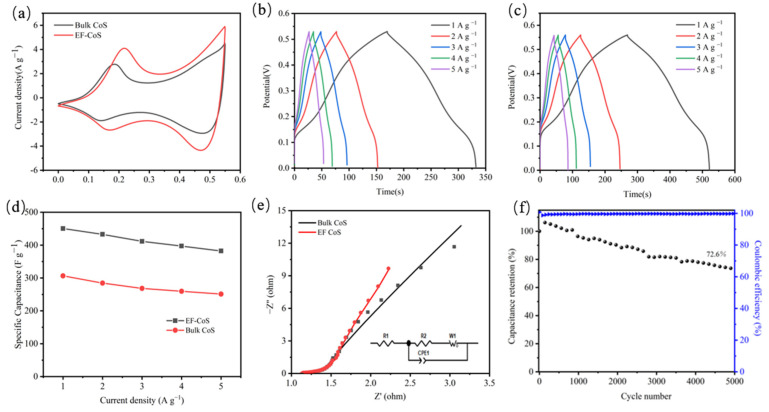
(**a**) CV curves of EF-CoS and bulk-CoS; GCD curves of bulk-CoS (**b**) and EF-CoS (**c**); (**d**) the corresponding specific capacitance calculated based on the GCD results of EF-CoS and bulk-CoS; (**e**) Nyquist plots of bulk-CoS and EF-CoS, and the chosen equivalent circuit; (**f**) Cycle performance and coulombic efficiency of the EF-CoS.

The electrochemical properties of bulk-CoS and EF-CoS sample as electrode materials as supercapacitors were investigated by a classical three-electrode system under aqueous condition (6 M NaOH aqueous solution). The CV curves of bulk-CoS and EF-CoS with a scan rate of 5 mV∙s^−1^ in the range of potential from 0 to 0.55 V are demonstrated in Figure 4a, which presents a similar shape of rectangular with evident oxidation and reduction peaks illustrating the capacitive characteristics of the electrodes mainly derived from the combined effect of Faraday redox reactions and double layer capacitance. The CV curves both exhibit two redox couples corresponding to the simultaneous reversible electron transfer processes between cobalt ions of different valence states (Co^2+^/Co^3+^ and Co^3+^/Co^4+^) on the surface of the *CoS* electrode in alkaline electrolyte media [26]:

*CoS* + *OH^−^*→*CoSOH* + *e^−^* (6)

*CoSOH* + *OH^−^*→*CoSO* + *H*_2_*O* + *e* (7)

The significantly higher peak current density of EF-CoS than bulk-CoS implies a more intense redox process on the electrode. Additionally, a bigger closed area under the CV curves of EF-CoS suggests greater charge storage capacity than bulk-CoS electrodes, which probably related to the fluffier structure and larger specific surface area after electrochemical exfoliation. Figure 4b,c present GCD curves of bulk-CoS and EF-CoS electrodes at different current densities, respectively. The identification of nonlinear charge–discharge curves further confirms that Faraday behavior is consistent with the CV measurements. The curves in both plots are remarkably symmetric when used to all current densities, demonstrating it is favorable reversibility for the Faraday redox reaction [27,28]. Furthermore, the resulting discharge curve is processed with Equation (3) to calculate the specific capacitance. Due to the remarkable increase in the discharge time of EF-CoS compared to bulk CoS, the capacitance enlarged from the original 307 F∙g^−1^ to 450 F∙g^−1^ at the current density of 1 A∙g^−1^, which unambiguously proves the electrochemically exfoliated EF-CoS electrodes offered larger capacity and superior charging and discharging performance. This is mainly caused by the fact that the active sites of sheet-like metal sulfides mainly exist at the edge positions, and the electrochemical exfoliation process can expose more sheets and more active sites, which is agreeable with the HRTEM outcomes discussed earlier. Furthermore, the rate performance (specific capacitance vs. current density) of EF-CoS and bulk-CoS under various current densities is summarized in Figure 4d. EF-CoS delivers high capacitance of 450, 433, 411, 397 and 382 F∙g^−1^ under the current densities of 1, 2, 3, 4 and 5 A∙g^−1^, respectively. In contrast, the capacitances of the EF-CoS electrodes used for comparison are 307, 285, 268, 260 and 251 F∙g^−1^ under the same current densities. It is found that with 5 times higher current densities, the obtained EF-CoS electrode maintains 84.7% capacitance while bulk-CoS maintained 81.9%, demonstrating that the exfoliated samples exhibit higher capacitance as well as a substantially improved rate capability property [29,30].

Electrochemical impedance spectra (EIS) were examined in the frequency range of 10^−2^ to 10^5^ Hz at the bias of 5 mV to better understand the electron/mass transport characteristics within the electrodes and between the electrode/electrolyte interfaces, as the results given in Figure 4e. The equivalent circuit model that is used to fit its EIS spectrum is shown in the inset of Figure 4e and the quantified impedance parameters of the fitting results are presented in Table 1. In a typical circuit, R_s_ stands for the equivalent electrolyte resistance, and the value of bulk-CoS and EF-CoS are similar because of the same electrolyte. R_ct_ represents the charge transfer resistance that related to the electronic conductivity of electrode materials and reveals the interfacial resistance of electrode and electrolyte, and the R_ct_ value of EF-CoS being lower than bulk-CoS indicates the easier faradaic reaction, which depends on the stronger conductivity of the exfoliated samples. The high electron conductivity of the electrode facilitates the charge transfer between the electrode and electrolyte interface, thereby diminishing the charge transfer resistance. CPE corresponding to a permanent phase element is employed to indicate the EDLC procedure instead of pure capacitance owing to the non-uniformity. The Wo as Warburg element (open) is adopted to illuminate the diffusion method, where W-R is the diffusion resistance and W-T is correlated with the diffusion length and diffusion coefficient of ion. W-R and W-T values of the EF-CoS electrode are distinctly lower than that of bulk-CoS, suggesting the superior ion diffusion efficiency of EF-CoS. Clearly, the results reveal the rapid migration of ionic and electrons within the EF-CoS electrodes and between the electrode/electrolyte interfaces [31,32,33].

Electrochemical stability is an additional critical indicator in determining the quality of supercapacitor electrodes for many practical applications. As shown in Figure 4f, capacitance retention and coulombic efficiency of electrochemically exfoliated EF-CoS for 5000 cycles at 10 A∙g^−1^ were used to evaluate cycling performance including. After 5000 continuous cycles, the specific capacitance is still 73% (263 F·g^−1^) of its initial value, which shows the electrode has favorable reversibility in the repeated charging and discharging. Additionally, the charge and discharge efficiency, also known as Coulomb efficiency, can be calculated using Equation (8):

(8) $$\eta =\frac{t_{d}}{t_{c}}\times 100\%$$

in which η is Coulomb efficiency, *t_c_* is the charging interval and *t_d_* is the discharging interval. The charge–discharge efficiency of the EF-CoS electrode was also recorded in Figure 4f and maintains a stable high value of 99.8% after 5000 continuous cycles. The promising stability demonstrates that there is a reversible surface redox interaction occurring between the electrolyte and EF-CoS electrode, affirming the stability of the EF-CoS electrodes as a promising active electrode material used in energy storage area.

Table 2 presents a comparative overview of the electrochemical responses of previously reported CoS-based supercapacitors. In conclusion, the findings suggested that the metallic EF-CoS sample with its unique structure, high specific capacitance and good stability would be a qualified candidate for eligible supercapacitor electrodes for practical applications.

**Figure 5:**
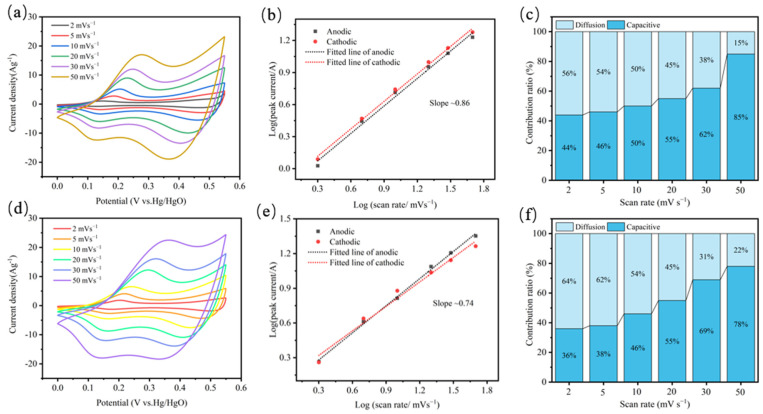
CV curves at different scanning rates of EF-CoS (**a**) and bulk-CoS (**d**); Linear fitting of the log (peak current) and log (scanning rate) for the anodic and cathodic peaks in the CV curves of EF-CoS (**b**) and bulk-CoS (**e**); Relative contribution of the capacitive and diffusion-controlled charge storage at different scan rates of EF-CoS (**c**) and bulk-CoS (**f**).

To further investigate the detailed electrochemical procedures of bulk-CoS and EF-CoS electrodes, the energy storage mechanism and dynamic characteristics of the electrode materials were analyzed using CV data. As the CV curves in Figure 5a,c show, the anodic and cathodic peaks potentials move in more positive and negative directions, respectively, and the peak current density magnifies in accordance with the scan rate from 2 to 50 mv∙s^−1^ on account of the limitation of ion diffusion. Figure 5b,d records a well linear relationship between the peak anode and cathode current densities (*i_p_*) and the squaring root of scan rates (*v*),which commonly obey the relationship as follows [35,38]:

*i_p_* = *av^b^* (9)

*log*(*i_p_*) = *blog*(*v*) + *log*(*a*) (10)

where *a* is a variable parameter and *b* is the slope of the curve. The *b* value of 0.5 represents that the reaction kinetics of the electrode is solely achieved by the diffusion-limited redox reaction, also known as battery type or pseudo-capacitance; the *b* value close to 1.0 denotes that the ideal capacitive behavior is mainly caused by the surface control process, also known as electrochemical double layer capacitance. Results from the peaks plot, the values of *b* for bulk-CoS and EF-CoS electrodes are 0.86 and 0.74, respectively, demonstrating that the capacitance of both electrodes derive from the mix of diffusion-controlled and surface-controlled capacitance process [39]. However, the *b*-value of EF-CoS electrodes decreases and is closer to 0.5, indicating that the charge storage process of EF-CoS tends to be dominated by more diffusion of the OH^−^ process of the battery-like behavior, corroborating the more intense ion insertion and removal processes of the EF-CoS electrode. The capacitance contribution to the total capacity can be further quantified using the following equation:

(11) $$i\left(V\right)=k_{1}v+k_{2}v^{\frac{1}{2}}$$

where *i* represents the current response at a fixed potential *V*, *k*_1_ and *k*_2_ are constants and *v* is the scan rate. By plotting *i* against *v*^1/2^, we can determine constants k_1_ and k_2_ and directly calculate the contribution of capacitance (*k*_1_*v*) and diffusion (*k*_2_*v*^1/2^) controlled processes. Figure 4c,f present the contributions of these two processes at varying scanning rates for the samples before and after exfoliation. As the scan rate increases and the time for ions to diffuse into the lattice decreases, the contribution of the diffusion-controlled process reduces. Notably, EF-CoS electrode exhibited a greater percentage of OH^−^ diffusion process control at the same scanning rate.

According to the above results, the electrochemical properties of EF-CoS are preferred to bulk-CoS, proving that the 2D structure is conducive to faster ion insertion/desorption and will ameliorate the electrochemical activity of EF-CoS. The more remarkable electrochemical properties of EF-CoS may have the following reasons: (1) Surface area effect. EF-CoS has a larger specific surface area for effective electrolyte penetration and interfacial reaction. (2) Nanoscale size effect. The layer-by-layer stacked structure of bulk-CoS caused the active sites deeply encapsulated and difficult to reach by ion diffusion, and the single or less layer nanosheets facilitate shorter diffusion paths for charge carriers. (3) High electron conductivity. The above EIS results indicate that the EF-CoS electrode possesses a smaller resistance and a larger diffusion coefficient [40,41].

**Figure 6:**
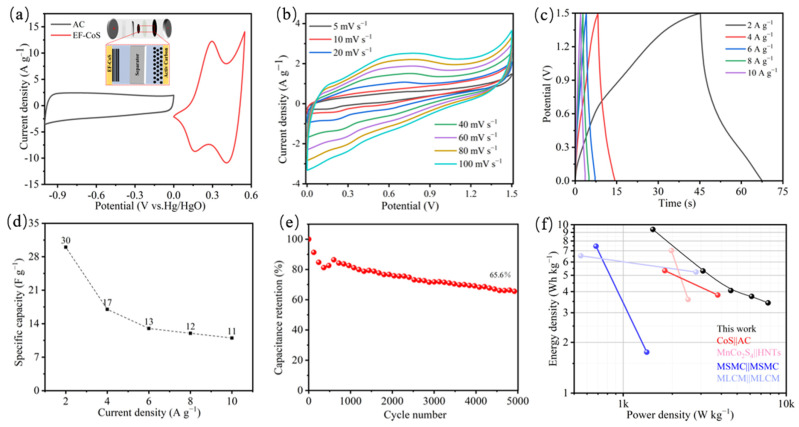
Electrochemical performance of assembled hybrid asymmetric supercapacitor. (**a**) CV curves of EF-CoS and AC electrodes performed in a three-electrode configuration and (inset) structure diagram of the EF-CoS//AC device; (**b**) CV curves of the EF-CoS//AC device at different scan rates; (**c**) GCD curves of the EF-CoS//AC device at different current densities; (**d**) Rate performance calculated based on the GCD (**e**) Cycle performance and coulombic efficiency of the EF-CoS//AC device measured at 1 A g^−1^; (**f**) Ragone plots of the EF-CoS//AC device compared with some other reported devices.

To further evaluate the potential of EF-CoS in practical applications, a BSH device (EF-CoS//AC) was assembled employing AC and 3 M KOH as anode materials and aqueous electrolytes, respectively, as shown in the inset of Figure 6a. The various voltage windows of EF-CoS cathode and AC anode in the three-electrode system can be observed by the CV curves in Figure 6a, from which the voltage windows of EF-CoS and AC are between −1.0–0 V and 0–0.55 V, respectively, showing that the BSH device should theoretically have a wide operating voltage of ~1.55 V. In order to obtain the appropriate voltage window for the assembled BSH, CV curves of the device were obtained in various voltage conditions range from 0–1.1 V to 0–1.7 V. As observed in Figure 5a, the total range is determined to be 0~1.5 V since it can be observed that the voltage window can remain stable under this condition. The CV curves of EF-CoS//AC device are illustrated in Figure 6b, whose similar CV curve shapes point to the excellent rate capability. Figure 6c presents GCD curves of a fabricated asymmetric supercapacitor under various current densities, from which could be calculated the specific capacitance of the EF-CoS//AC device decreases from 30, 17, 13, 12 to 11 F∙g^−1^ with the discharge current density aggrandizes from 2, 4, 6, 8 to 10 A∙g^−1^. Additionally, the stability of the EF-CoS//AC device was also obtained by charging the electrode with the current density of 3 A∙g^−1^, and it showed favorable cycling stability, maintaining 66% capacitance retention after 5000 cycles of charge and discharge, as demonstrated in Figure 6e. The Ragone plot, the corresponding power density and energy density of the as-assembled EF-CoS//AC device, can be found in Figure 6f. Based on Equations (4) and (5), the energy density of EF-CoS//AC hybrid device is 9.4 Wh∙kg^−1^ at the power density of 1520 W∙kg^−1^ and maintained at 3 Wh∙kg^−1^ even at 7550 W∙kg^−1^. These outperform some other cobalt-based BSH devices fabricated by CoS//AC (15.58 Wh∙kg^−1^ at 700.12 W∙kg^−1^) [34], CoS//AC (5.3 Wh∙kg^−1^ at 1800 W∙kg^−1^) [36], an MnCo2S4/HNTs symmetric device (6.98 Wh∙kg^−1^ at 1970.6 W∙kg^−1^) [42] and MSMC//MSMC device (7.41 Wh∙kg^−1^ at 160 W∙kg^−1^) [43].

## 4. Conclusions

In conclusion, we demonstrate that the layered metallic CoS bulk with high electrical conductivity can be electrochemically exfoliated into 2D CoS nanosheets and prove their applicability to the area of energy storage technology. The results of characterization showed that the phase of the metallic CoS before and after exfoliation basically remained, but the morphology and thickness of the samples varied, obviously, and the specific surface area increased. The unique 2D sheet structure shortens the ions transport path and exhibits better conductivity and high specific capacitance of 450 F∙g^−1^ at 1 A∙g^−1^. Meanwhile, high-quality EF-CoS displayed superior rate performance of capacitance retention rate of nearly 85% when the current density expanded to five times, and good cycling stabilization of 73% of the original capacity after 5000 cycles. The ultracapacitor performance of EF-CoS is significantly improved compared with bulk-CoS, attributed to the faster charge transfer across the EF-CoS electrode and electrolyte interface. The fabricated EF-CoS//AC device has a specific capacitance of 30 F∙g^−1^ at 2 A∙g^−1^, and the energy density of 9.4 Wh∙kg^−1^ when the power density is 1520 W∙kg^−1^. This work illustrated the potential application of layered metallic transition metal sulfides as high-performance energy storage devices.

## Figures and Tables

**Table 1:** Equivalent circuit parameters of the electrodes fabricated with the bulk-CoS and EF-CoS.

Samples	R_s_ (Ω)	R_ct_ (Ω)	CPE-T (F)	CPE-P	W-R (Ω)	W-T	W-P
Bulk-CoS	1.18	0.13	0.24	0.81	0.0848	0.5833	0.49
EF-CoS	1.16	0.06	0.06	0.78	0.0024	0.0028	0.26

**Table 2:** The comparison of various capacitive performances.

Electrode Materials	Current Density (A g^−1^)	Capacitance (F g^−1^)	Retention (Cycles)	Ref.
flower-like CoS	0.5	357	87.3% (2000)	[7]
flower-like CoS	1	348	97.2% (1000)	[34]
Co_9_S_8_ nanoparticles	1	502	87% (7000)	[35]
dumb-bell shaped CoS	5	310	95% (5000)	[36]
Co_9_S_8_@S, *n*-doped carbon	1	429	98% (2000)	[37]
flower-like CoS	1	409.3	90% (1000)	[11]
fungus-like CoS	1	350.4	88.4% (10,000)	[27]
EF-CoS	1	418	72.6% (5000)	This work

## Data Availability

Not applicable.
